# MicroRNAs and lung cancer: Biology and applications in diagnosis and prognosis

**DOI:** 10.4103/1477-3163.67074

**Published:** 2010-08-03

**Authors:** Reema Mallick, Santosh Kumar Patnaik, Sai Yendamuri

**Affiliations:** 1 Northeastern Ohio Universities College of Medicine, Rootstown, OH, USA; 2 Department of Thoracic Surgery, Roswell Park Cancer Institute, Buffalo, NY, USA; 3 Department of Surgery, State University of New York at Buffalo, Buffalo, NY, USA

**Keywords:** Carcinogenesis, gene expression, microRNA, lung cancer

## Abstract

MicroRNAs are tiny non-coding RNA molecules which play important roles in the epigenetic control of cellular processes by preventing the translation of proteins from messenger RNAs (mRNAs). A single microRNA can target different mRNAs, and an mRNA can be targeted by multiple microRNAs. Such complex interplays underlie many molecular pathways in cells, and specific roles for many microRNAs in physiological as well as pathological phenomena have been identified. Changes in expression of microRNAs have been associated with a wide variety of disease conditions, and microRNA-based biomarkers are being developed for the identification and monitoring of such states. This review provides a general overview of the current state of knowledge about the biology of microRNAs, and specific information about microRNAs with regard to the diagnosis and prognosis of lung cancer.

## INTRODUCTION

Lung cancer is both the most common type of cancer as well as the most common cause of cancer-related death worldwide.[1] In the United States, an estimated 220,000 new cases and 159,000 deaths from the disease were expected in 2009, accounting for 15% of all new cancer cases and 28% of all cancer deaths, respectively.[2] Surgical resection is the optimal treatment for lung cancer, the most common (70%) variant of which is non-small cell lung cancer (NSCLC).[3] For those in the earliest stage of NSCLC (stage IA), the five-year survival rate is only 73%.[4] That the five-year survival rate of lung cancer patients is only 15% highlights the importance of a better understanding of lung cancer biology to improve prevention, diagnosis, and treatment of this disease. MicroRNAs form a relatively new class of molecules whose importance in this regard is the focus of a large body of research.

MicroRNAs are ultrashort (18–25 nucleotides), single-stranded RNA molecules that do not encode proteins but instead restrict the production of proteins by inhibiting translation from coding or messenger RNAs (mRNAs), or by causing their degradation. Their role as epigenetic mediators underscores the importance of the non-coding portion, the “dark matter,” of the genome as well as of small RNAs which also include small interfering RNAs (siRNAs) and Piwi-interacting RNAs (piRNAs).[5] Since the discovery of the first microRNA, *lin- 4*, in the worm *Caenorhabditis elegans*,[6] more than 850 microRNAs have been identified in humans, and thousands of microRNAs have been discovered in other animals, plants, and viruses. Novel microRNAs are continually being discovered as investigators explore sequences of small RNAs in more types of cells using improved techniques.[7] A large number of studies have been undertaken on the biology of microRNAs, microRNA alterations and their effect on physiological and pathological processes, and the potential utility of microRNA-based science in the clinical arena. This review summarizes the current state of knowledge in this rapidly evolving field to aid investigators understand and evaluate information emerging from the field of microRNA research.

## BIOGENESIS OF MICRORNAS

**Figure 1:**
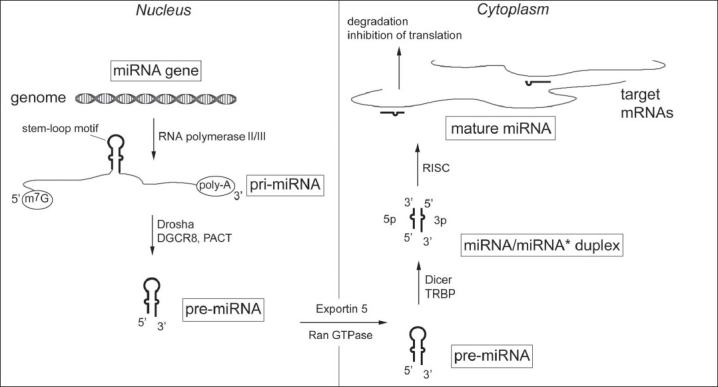
Biogenesis and mechanism of action of microRNAs. MicroRNAs are typically generated by RNA polymerases from microRNA-encoding genes as primary microRNA transcripts (pri-miRNAs) bearing stem-loop structural motifs, 5’ caps and poly-A tails. The Drosha endoribonuclease with associated RNA-binding proteins such as DGCR8 and PACT then removes the stem-loop regions from the pri-miRNAs to generate 60-70 nucleotide-long, two nucleotide-long 3’ overhang-bearing precursor microRNAs (pre-miRNAs) which are transported out of nucleus to the cytoplasm by the activity of Ran GTPase and Exportin 5 transporter proteins. The Dicer ribonuclease, working with RNA-binding proteins such as TRBP, then cleaves the loop region from the pre-miRNAs to generate the miRNA/miRNA^*^ RNA duplex that has two, 19-25 nucleotide-long, partially complementary, single-stranded RNA molecules (5p and 3p). The duplex is loaded onto the RISC multiprotein complex, where one of the two single RNA strands is degraded, and the other RNA, the mature microRNA, is left to guide the complex to microRNA-specifi c on target mRNAs to cause their degradation or inhibit the translation of proteins from them. MicroRNA binding to the target mRNAs requires only partial sequence complementarity overall, but complete complementarity in the seed region of the mature microRNA is believed to be required.

Figure 1 illustrates the canonical pathway for microRNA biogenesis. Most microRNAs are generated from stem-loop secondary structural motif-bearing precursor RNA molecules (pre-microRNAs) which in turn are generated from primary RNA molecules (pri-microRNAs) transcribed by the RNA polymerases II[8] or III[9] in the nucleus. Pri-microRNAs are much longer RNAs and are the transcripts of microRNA genes. Like mRNAs, they possess a 5’ cap and a 3’ poly-A tail. Pre-microRNAs, which bear two nucleotide-long 3’ overhangs, are cleaved off pri-microRNAs by the action of the “microprocessor,” a protein complex of the type III nuclear endoribonuclease, Drosha, and the double-stranded RNA-binding protein, DGCR8 (DiGeorge syndrome critical region gene 8; also known as Pasha). Pre-microRNAs are 60–70 nucleotide-long and retain the stem-loop secondary structural motif. In the case of a small number of microRNAs, pri-microRNAs are instead generated as introns (“miRtrons”) that are spliced from RNA transcripts generated from non-microRNA genes.[10] Pre-microRNAs are transported out of the nucleus by the Ran GTPase-dependent nuclear transporter protein, Exportin 5.[11] In the cytoplasm, they are acted upon by the type III endoribonuclease, Dicer, which cleaves their loop to release the duplex RNA stem as an 18–25 base-pair-long double-stranded RNA.[12]

The duplex RNA, also referred to as the miRNA/miRNA* duplex, is incorporated in an ATP-dependent fashion into the RNA-induced silencing complex (RISC), a multi-protein assembly, that also processes the short double-stranded RNA molecules siRNAs and piRNAs that, like microRNAs, are also responsible for the phenomenon of RNA interference (RNAi). The RISC complex comprises of proteins such as the Argonaute proteins (Ago 1 to 4 in humans), some of which possess endoribonuclease activity,[13] and TRBP (trans-activating RNA-binding protein; also known as Loquacious or Loqs),[14] and PACT (also known as protein activator of PKR kinase),[15] which are double-stranded RNA-binding proteins. Unlike siRNAs, the two strands of the microRNA duplex are imperfectly complementary. One strand of the duplex (“guide strand”) becomes the “mature” microRNA; the other one (“passenger strand”) is degraded by the endoribonuclease activity of the Ago 2 protein.[16] Either of the two strands of the duplex can become the guide strand. RISC is guided to the target mRNA molecules by the mature microRNA loaded on it and causes the degradation of the mRNAs or the inhibition of translation from them.[17] The RISC proteins, microRNA, target mRNAs, and other proteins involved in degradation of RNAs localize in cytoplasmic structures called as P or GW bodies.[18,19]

Mature microRNAs can undergo further modifications such as 3’ uridylation or adenylation, and nucleotide substitution.[20] Such modifications can affect their stability as well as function. Identification and characterization of trans-acting proteins, such as the XRN2 exoribonuclease which affects microRNA stability by degrading mature microRNAs, is an important area of current microRNA research.[21]

## NOMENCLATURE OF MICRORNAS

New microRNA sequences are typically deposited in the miRBase microRNA registry[22] which assigns names to microRNAs. MicroRNA names have a prefix indicative of the species and a unique numerical suffix. For example, *hsa-miR-21* refers to the mature *miR- 21* microRNA of humans, whereas *mmu-miR-21* refers to the corresponding murine ortholog. Mature microRNAs whose sequences differ at only one or two nucleotide positions are assigned the same numerical suffix along with a distinguishing alphabetical identity. For example, the mature microRNAs *hsa-miR-146a-5p* and *hsa-miR-146b-3p* are the products of different genes located on different chromosomes but differ from each other at only two nucleotide positions. Mature microRNAs that have an identical sequence but arise from different genes are given additional numerical suffices. For example, *hsa-miR-1-1* and *hsa-miR-1-2*, whose genomic loci are on chromosomes 20 and 18, respectively. Each of the two strands of the duplex RNA stem (miRNA/miRNA* duplex; Figure 1 released by Dicer from pre-microRNAs can be independent mature microRNAs, and they are denoted with either a “5” or “3” suffix that refers to their 5’ or 3’ location in a pre-microRNA molecule. For example, the pre-microRNA for *hsa-miR-30a* gives rise to the *hsa-miR-30a-5p* and *hsa-miR-30a-3p* mature microRNAs. When one of the 5p and 3p variants of a microRNA is more abundant, the other is sometimes referred to with a “*” suffixed; thus, *hsa-miR-146b-5p* may simply be called *hsa-miR-146b*, and, its less abundant partner, hsa-miR-146b-3p, hsa-miR-146b*

## MICRORNA TARGETS

MicroRNAs affect the translation of proteins from mRNAs by inhibiting it or by causing the degradation of the mRNA molecules. Different microRNAs target different mRNAs, and the target specificity is determined by sequence complementarity between the microRNA and mRNA molecules. Each species of microRNA is believed to target hundreds of different mRNAs, and an mRNA may be targeted by different microRNAs. Clearly, the effect of alteration in the expression of a single microRNA in a cell can depend on its concentration as well as on the concentration of the mRNAs that are targeted by it.

Studies have shown that targeting does not require a perfect sequence complementarity between the microRNA and a target mRNA as long as a six nucleotide-long region at the 5’ end of the microRNA (positions 2–7 ), also known as the “seed” region, binds perfectly to the mRNA[23][Figure 1]. Functional microRNA-binding sites were initially thought to exist only in the 3’ untranslated region (UTR) of the target mRNAs, but it has been shown that such sites can be present in the 5’ UTR and even the coding regions of target mRNAs. A number of computational approaches have been developed to predict target mRNAs and identify microRNA binding sites on them.[24–26] Some of the approaches consider the 3’ UTR of mRNAs as the only possible microRNA-binding region, and some require conservation of microRNA-binding site sequences in orthologous mRNAs from multiple species.

Confirmation that an mRNA predicted to be a microRNA target is indeed so is provided by showing an inverse correlation between the level of the microRNA and the level of the protein translated from the mRNA or of the mRNA itself. Assays in which reporter genes such as those encoding for luciferase or fluorescent proteins engineered to have specific microRNA-binding sites are also used for this purpose. High-throughput approaches for genome-wide identification of target mRNAs, such as Ago-IP[27] and RIP-ChIP,[28] are being actively developed.

Some studies have suggested that microRNAs may directly influence the transcription of genes.[29,30] For example, *miR-10a* has been shown to induce inhibition of transcription from the *HOXD4* gene by associating with the promoter region of the gene and causing its methylation.[31]

## EXTRACTION AND QUANTIFICATION OF MICRORNAS

Commonly-used RNA extraction methods, such as those using phenol and chloroform, Trizol™ reagent, and silica-containing spin-columns, are also usable with minor modifications for the extraction of small RNAs such as microRNAs from individual cells, tissues, or fluids. Unlike mRNAs, microRNAs have been shown to be preserved remarkably well in formalin-fixed and paraffin-embedded (FFPE) tissue, and this has obviated the need for fresh frozen tissue for microRNA expression studies.[32]

A cell can lack a particular species of microRNA, or it may carry a few to several thousands of copies of it. General techniques used for quantification of RNA, such as Northern blotting, reverse transcription-PCR (RT-PCR), RNA:RNA *in situ* hybridization, and microarray analysis, are also applicable for measurement of mature as well as precursor microRNAs. Cloning into an RNA library and sequencing,[33] single molecule sequencing,[34] surface-enhanced Raman spectroscopy,[35] surface plasmon resonance spectroscopy,[36] nanomechanical sensing,[37] and single-silver nanoparticle counting[38] are some other techniques being developed to detect and quantify microRNAs. The short length as well as sequence similarities of microRNAs does necessitate modification of such techniques, for example, through the use of stem-loop primers for reverse transcription,[39] and of locked nucleic acid-containing probes on microarrays.[40]

An important issue with regard to microRNA quantification is that of normalization. Unlike for mRNAs, there are no established housekeeping microRNAs which can be used for this purpose. Other, non-microRNA small RNAs such as the *RNU6B* nuclear RNA (also known as *U6B*) have therefore been routinely used for normalization purposes.[41] In this context, it should be noted that RNAs longer than 100 or so nucleotides, which include most mRNAs, may be lost if RNA is extracted from biological material following certain protocols. Also, degraded non-microRNA RNA can appear in microRNA fractions and confound normalization of quantification based on the starting amount of RNA. In the absence of well-accepted normalization candidates, establishment of experiment-specific normalization parameters thus becomes important.

## MICRORNAS IN BODY FLUIDS

Recent studies have shown that microRNAs can also be identified in serum or plasma. This finding has the potential to enhance the utility of microRNAs as biomarkers to diagnose, prognosticate, and monitor lung cancer via non-invasive methods. It is believed that tumor cells secrete cell membrane-enveloped microvesicles termed exosomes into the extracellular space that can eventually be detected in the blood.[42–44] These exosomes are 30–100 nm in size and contain proteins, cytosol, mRNA, and microRNAs, but are believed to lack DNA, nuclear RNA such as *RNU6B* and small nucleolar RNAs, and large, ribosomal RNA.[43,45,46] Their lipid membrane protects them from the circulating ribonucleases present in blood,[45,47] and microRNAs have been shown to be stable in serum stored at 4 °C for up to 4 days.[48] It should be noted that since microRNAs are released into circulation by non-cancer tissue as well, because of cell lysis or through secretion of exosomes, serum microRNA profiles may[48] or may not[46] correspond to cancer-tissue profiles.

Chen *et al*. reported that serum RNA concentration averages about 100 ng/ml and, by employing a deep sequencing methodology, showed that most of the RNA isolated from serum is less than 33 nucleotides-long, with most of them containing 21–23 nucleotides, thus suggesting an enrichment of the microRNA fraction in serum RNA.[47] Compared to healthy subjects, sera from NSCLC cases (*n* = 11) had 28 microRNAs missing and 63 new microRNAs present. Using quantitative RT-PCR on RNA isolated from sera of 152 cases of lung cancer and 75 healthy subjects, they further confirmed that microRNAs *miR-23* and −*225* are significantly elevated in the former. In a study on 303 NSCLC patients, the same group has shown that levels of *miR-486, −30d, −1*, and *−499* are significantly associated with overall survival.[49]

Because exosomes can bear cell-surface proteins characteristic of the cell of origin, one may be able to isolate exosomes in the blood that have originated from specific tissue. Using an antibody against epithelial cell adhesion molecule (EpCAM), Rabinowits *et al*. enriched exosomes secreted by the respiratory epithelium into plasma in 27 cases of NSCLC (American Joint Committee on Cancer stages 1–4 ) and compared the levels of 12 microRNAs in the exosomes against those in nine healthy individuals by RT-PCR. MicroRNAs *miR-17-3p, −21, −106a, −146, −155, −191, −192, −203, −205, −210, −212*, and *−214*, whose levels are known to be altered in NSCLC tissue, were also elevated in EpCAM-bearing plasma exosomes in the NSCLC cases. Interestingly, the lung cancer patients had both elevated RNA (160 ng/ml vs. 70 ng/ml) and exosomes (2.9 mg protein/ml vs. 0.8 mg protein/ml), suggesting that tumor cells secrete more exosomes into the extracellular medium.[42]

Besides blood, microRNAs have been detected in other body fluids such as saliva, urine, and semen. It is believed that, similar to the case with blood, they are present within exosomes and are thus more resistant to degradation by ribonucleases in the fluids. The utility of microRNAs in bodily fluids as biomarkers for oral cancer,[50] bladder cancer,[51] etc., is being actively investigated. MicroRNAs can also be quantified in sputum, and in a study of 23 cases of NSCLC (stages I–IV) patients and 17 controls, increased levels of microRNA *miR-21* relative to *RNU6B* were associated with cancer with sensitivity and specificity values of 70% and 100%, better than the values of 48% and 100%, respectively, that were seen with sputum cytology.[41] Values of 81% and 92%, respectively, were seen with a microRNA signature made of *miR-21, −486, −375*, and *−200b* in a study of 112 cases of lung adenocarcinoma.[52]

Important aspects of quantification of microRNAs in body fluids for microRNA are the relatively low amount of RNA in them, and the lack of a suitable RNA for normalization purpose. Housekeeping RNAs such as *RNU6B* and *GAPDH* are usually not present in the RNA fraction of body fluids.[53] Studies quoted above used different normalization methods. Some used the starting fluid volume, whereas others used the starting RNA amount. Extensive validation using independent sample-sets, data demonstrating replicability of quantification, and the use of clinically relevant controls are important attributes of design of studies that explore the clinical utility of microRNA-based biomarkers.

## MICRORNA ALTERATIONS IN LUNG CANCER

Variations in expression of microRNAs in cancer was first shown by Calin *et al*. for chronic lymphocytic leukemia.[54] Such cancer-specific changes have since been shown for a variety of cancers, and microRNA expression profiling can be used to accurately identify tissue of origin for both primary and metastatic cancers.[55] Unlike what is often seen with mRNA or gene expression studies, a particular characteristic of such microRNA expression changes is that the degree of change is much smaller (usually in the 1.2–2.5 fold change range). This suggests that small changes in microRNA expression can have significant effects on the phenotype, which is consistent with the known ability of microRNAs to simultaneously influence the expression of hundreds of genes. Although oncogenic or tumor suppressive roles for specific microRNAs, such as the tumor suppressor *miR-15a* in chronic lymphocytic leukemia,[54] have been identified, it is believed that most of the alterations of microRNA expression seen in cancer tissues just reflect the cancer phenotype of the cells. It should be noted that possibly because of variations in microRNA profiling methodologies, analytic approaches, sample-sizes, population types, methods of tissue archival, RNA extraction, etc., the nature of microRNA alterations identified in similar studies can be dissimilar. Tumor tissue contains non-cancerous cells such as those in its stromal compartment which respond to as well as affect cancer cells, and changes in microRNA levels detected by cancer tissue profiling may not reflect changes that occur within the cancer cells. Expression profiling using laser capture microdissected tissues can be used to identify cancer cell-specific microRNA changes more accurately.[56]

Changes in microRNA expression in lung cancer have been extensively studied. In microarray-based expression analysis of 352 microRNAs in 104 pairs of fresh-frozen lung cancer (of different histology and stage) and normal adjacent tissue, Yanaihara and colleagues identified 43 microRNAs that were differentially expressed.[57] MicroRNAs that were overexpressed in lung cancer included *miR-21, −191, −210, −155, −205, −17−3p, −214, −212, −106a, −192, −197, −146, −203*, and *−150*; downregulated microRNAs included miR-126*, −143, −224, −126, −30a − 5p, −149, −198, −145, −95, −218, −124a, −27b, −32, −29b − 2, −220, −33, −101 − 1, −125a, and −124a−3. In a similar study in which fresh-frozen samples from a smaller number of lung cancer cases were examined by microarray for expression of 190 microRNAs, 35, and three microRNAs were found to be up- and downregulated, respectively, in cancer.[58] Some of the affected microRNAs, such as *miR-17, −20, −210, −213, and −155*, were also found to be altered in cases of breast cancer in the same study. On the other hand, expression of *miR-218* was not affected in either cancer despite being universally downregulated in cancers of colon, stomach, prostate, and pancreas. Expression of microRNAs *miR-15a* and *-21* has also been noted to be down in both squamous cell carcinomas and adenocarcinomas of the lung.[56] In a study of 19 lung cancer cell-lines, expression of microRNAs belonging to the miR-17 −92 family of microRNAs was noted to be upregulated in lung cancer, especially those of the small cell variety.[59] Seike and colleagues examined the expression of 389 microRNAs in cancer tissue compared to normal lung in 28 cases of lung cancer among non-smokers by microarray hybridization and identified *miR-21, −141, −210, −200b* and *−346*, and miR-126*, −126, −30a, −30d, −486, −129, −451, −521, −128, −30b, −30c, −16a and −520, respectively, as up- and downregulated microRNAs.[60] In a similar study but of only adenocarcinomas in which the expression of 470 microRNAs was quantified, miR-126* and −145 were found to be downregulated in cancer whereas expression of *miR-21, −182, −183*, and *−210* was upregulated (fold changes of 2.7–11.2). Navarro and colleagues examined expression of 18 microRNAs in 33 paired fresh-frozen lung cancer-normal lung samples and found that the expression of all members of the *let-7* microRNA family except *let-7e*, and *miR-222* and *−155* were downregulated whereas that of microRNAs of the miR-17-92 cluster, *miR-221, −7e*, and *−98* was up-regulated.[61] Raponi *et al*. examined the expression of 328 microRNAs in fresh-frozen squamous cell carcinoma tissues and found 15 microRNAs that were differentially expressed in the disease, with upregulated microRNAs being *miR-210, −200c, −17−5p, −20a, −203, −200a, −106b, −93, −182, −83, −106a, −20b* and *−224* (1.2–2 log_2_ -fold change), and downregulated microRNAs being *let-7e* and *miR-200a* (0.4–1.3 log _2_-fold change).[62]

Subtypes of lung cancer too have characteristic microRNA expression. Higher expression of *miR-106a* has been associated with the small cell variant of lung cancer.[61] Lebanony and colleagues, in a study analyzing the expression of 141 microRNAs in FFPE tissue from 60 adenocarcinomas and 62 squamous cell carcinomas, have shown that level of *miR-205* relative to that of *miR-21* can be used to distinguish the two NSCLC sub-types with 96% sensitivity and 90% specificity.[63] Expression of *miR-205* in FFPE specimens, as detected by RT-PCR, was also used in a different study to successfully classify all 102 cases of NSCLC as squamous cell carcinoma or adenocarcinoma.[64] In yet another study, 34 of 440 microRNAs in 205 RNA samples from FFPE specimens analyzed by microarray were found to have significantly different expression between the two NSCLC histological subtypes.[65] Lung carcinoids have been shown to have a microRNA expression pattern distinct from that of other lung cancers and characterized by higher expression of *miR-94*.[55] Changes in microRNA expression during progression from hyperplasia to invasive squamous cell carcinoma of the lung cancer have been studied for 365 microRNAs by RT-PCR.[66] Sixty-nine microRNAs were found to be differentially expressed during the course of the disease; expression of *miR-34c, −15a*, and *−32* decreased progressively, whereas that of *miR-139* and *−199a* was step-specific.

Consistent with the above data and the known etiological relationship between smoking and lung cancer, smoking has been shown to alter microRNA expression in rodents[67] as well as humans.[65,68]

## PROGNOSIS OF LUNG CANCER AND MICRORNAS

Several studies have demonstrated the ability of microRNA expression profiling to predict the outcome of lung cancer patients after surgical resection. In Raponi *et al*.’s study of microRNA expression in squamous cell carcinoma tissues, reduced expression of microRNA *miR-146b* was associated with reduced overall survival following surgical resection of the tumor with an accuracy of 78% and a hazard ratio (HR) of 5.9 (95% confidence interval [CI]: 2.2–13.1).[61] HR associated with expression of *miR-155* was 2.3 (95% CI: 1.0–5.6). Association of *miR-155* levels with reduced overall survival in a study of 55 cases of lung cancer treated by surgical resection was also noted by Yanaihara *et al*., with a relative risk of 3.0 (95% CI: 1.1–8.1).[57] Levels of microRNA *let-7a-2* were also found to be prognostic in the study (relative risk of 0.95). Yu *et al*. assessed the expression of 157 microRNAs in fresh-frozen cancer tissue from 101 patients of stages I–III NSCLC by RT-PCR, and identified a five-microRNA signature (comprising of microRNAs *let-7a* and miR-221, −137, −372, and −182*) predictive of overall survival with an HR of 2.8 (95% CI: 1.1–7.0) and of relapse-free survival with an HR of 2.4 (95% CI: 1.1–5.1). Prognosticative value of *let-7* microRNA expression in 159 cases of stages I–III NSCLC (HR of 2.8 [95% CI: 1.6–4.9]) had been noted previously by Takamizawa and colleagues,[69] though such correlation of *let-7* expression with prognosis in a study of 81 cases of bronchioloalveolar carcinoma and adenocarcinomas was not observed in a different study.[70] Patnaik and colleagues examined the expression of 752 microRNAs in FFPE tissue surgically resected from 77 cases of stage I NSCLC and showed that microRNA expression pattern can prognosticate recurrence of the disease following surgery with as high an accuracy as 83% with associated HR of 9.0 (95% CI: 4.4–18.2).[71] In a study of 205 NSCLC cases that profiled 440 microRNAs in FFPE tissue using microarrays, a signature consisting of reduced expression of microRNAs *let-7e*, and *miR-34a, −34c−5p, −25*, and *−191* was found to be prognostic of poor survival among male smokers with stages I–IIIA squamous cell carcinoma, with HRs varying from 0.3 to 0.5 for the individual microRNAs.[65]

Although such correlations between prognosis and microRNA expression have been noted in multiple studies, causal linkages underlying such phenomena have yet to be identified. Also, many of the identified microRNA-based predictors have yet to be independently validated. While similar predictors have also been developed using mRNA expression data, the ability to use FFPE tissue provides a distinct advantage for clinical applicability and the ease of performing retrospective exploratory studies.

Chemosensitivity prediction using microRNA expression profiling is the focus of active investigation by several research groups. In microRNA profiling studies of NSCLC cell-lines, varyingly resistant to apoptosis induced by tumor necrosis factor (TNF)-related apoptosis-inducing ligand (TRAIL), *miR-221, −222, −100, −125b*, and *−15b*, and *miR-9* and *−96*, were down- and upregulated, respectively, in the resistant cell-lines.[72] Loss of heterozygosity for microRNA *miR-128b*, seen in 55% of NSCLC cases, has been correlated with improved clinical response and survival following treatment with gefitinib.[73]

## *IN VITRO* EXPERIMENTAL STUDIES

Observations from a number of experimental studies performed on cell-lines *in vitro* have suggested mechanistic roles for microRNAs in the lung cancer cell phenotype. A few examples are described here. MicroRNA *miR-7* has been shown to target epidermal growth factor receptor protein (EGFR, also known as HER1 or ErbB-1) and reduce activation of Akt and extracellular signal regulated kinase 1/2 using cell-lines.[74] On the other hand, EGFR-mutant cell-lines have been shown to have increased microRNA *miR-21* expression, and inhibition of *miR-21* has been shown to induce apoptosis in such cell-lines.[60] MicroRNA *miR-145* has been shown to inhibit growth of lung cancer cells *in vitro*, with the inhibition being stronger in cells with EGFR mutations.[75]

MicroRNAs *miR-29a, −29b*, and *−29c* have been shown to target methyltransferases DNMT *3a* and *3b* which affect the transcription of genes like *FHIT* and *WWOX*.[76] MicroRNA *miR-126* targets the CRK oncogene, and overexpression of the microRNA inhibits adhesion, migration, and invasion of cells *in vitro*.[77] MicroRNA *miR-126* is also known to target *VEGF-a* mRNA, and its overexpression in A549 lung cancer cells reduces their proliferation and tumorigenicity.[78] MicroRNA *miR-133b* targets members of the BCL2 family of pro-apoptotic mRNAs, *MCL1* and *BCL2 L2*.[79] In transgenic mouse models of lung adenocarcinoma, transfection of microRNAs *miR-34c, −34b, −145*, or *−142−5p*, known to be downregulated in lung cancer, has been shown to reduce lung cancer cell growth.[80] MicroRNAs *miR-221* and *222* are known to target cyclin D kinase inhibitor p27 and tyrosine kinase kit,[72] and by targeting PTEN and TIMP3 tumor suppressors, induce TRAIL resistance and enhance cellular migration through activation of the Akt pathway and metallopeptidases.[81] Overexpression of *miR-137, −372*, or −182* leads to increased invasiveness of lung cancer cell lines invasiveness, whereas that of *221* reduces their invasiveness.[82] Overexpression of *let-7a* or *-7f* in A549 lung cancer cells results in reduced colony formation.[69]

As indicated in the examples above, cell culture-based experiments have provided explanations for the observations seen in various clinical microRNA profiling studies. However, sometimes such experiments do not yield a satisfactory answer. For instance, no effect of overexpression of microRNAs *miR-30a* or *−191*, microRNAs that are downregulated in lung cancer, could be discerned in A549 lung cancer or BEAS - 2B normal bronchial epithelial cells.[83] Such results are seen possibly because many experiments do not incorporate the effects of the tumor microenvironment, or because changes in expression of certain microRNAs in cancer tissue actually arise from changes in the non-cancerous stromal compartment of the tissue.

## SEQUENCE POLYMORPHISMS AMONG MICRO RNAS AND TARGET MRNAS

An emerging paradigm is the role of polymorphisms of microRNAs and mRNAs in influencing the stability of microRNAs as well as the interaction between microRNAs with their mRNA targets. Polymorphisms or mutations in genes can alter their susceptibility to regulation by microRNAs, and this can allow cancer cells to bypass normal microRNA regulation. Tian and colleagues have shown an odds ratio of association of a particular CC polymorphism in microRNA *miR-196a-2* with lung cancer of 1.3 (95% CI: 1.0–1.6).[84] The CC genotype was shown to increase levels of mature *miR-196a* but not of *pre-miR-196a*, suggesting that the sequence variation affected microRNA processing post-transcriptionally.[85]

Chin and colleagues studied sequence variations in the ten *let-7* microRNA binding sites in the 3’ UTR of the *KRAS* gene in a cohort of 54 NSCLC cases. Variations were found in 18–20% of lung cancer cases as compared to 6% for world population, suggesting a relative risk of 1.4–2.3, and *in vitro* transfection experiments showed that variant *KRAS* alleles resulted in increased KRAS protein expression in A549 lung cancer cells.[86]

## THERAPEUTIC IMPLICATIONS OF MICRORNAS

Specific microRNA expression can be knocked down using antisense oligonucleotides, or enhanced by virus-based gene therapy or administration of synthetic microRNA analogues. Although such approaches have yet to be tested in humans, promising preliminary results have been seen in animal studies. For instance, intratumoral injection of synthetic *let-7* microRNA in a mouse model of lung cancer has been shown to reduce tumor burden.[87] Unlike siRNAs, only a few microRNA-based therapeutic strategies are under investigation, possibly because the promiscuity of microRNAs in targeting scores of different mRNAs means that artificially affecting levels of a single microRNA can have undesirable effects. Nevertheless, our understanding of the biology of microRNAs, specifically, the pathways and mRNAs affected by those whose expression is altered in diseased states, can help with the identification of new molecular targets for therapy.

## CONCLUSION

MicroRNAs are short non-coding RNAs that can restrict the expression of proteins from mRNAs. The ability of a single microRNA to influence the expression of several hundred different mRNAs can help identify novel biological pathways. Characterization of variations in microRNA levels in diseased tissue, as well as the stability of microRNAs in FFPE specimens and biological fluids, makes microRNAs promising biomarkers, and can lead to the development of novel diagnostic and therapeutic approaches for lung cancer.

## AUTHOR’S PROFILE

**Ms. Reema Mallick** is a final-year medical student at the Northeastern Ohio Universities College of Medicine in Rootstown, Ohio, USA. Over the last year she has conducted research on serum microRNAs and on the biology of microRNAs.

**Dr. Sai Yendamuri** is an Assistant Professor in the Department of Thoracic Surgery at the Roswell Park Cancer Institute (RPCI), and in the Department of Surgery at the State University of New York in Buffalo, New York, USA. He is the director of the Thoracic Oncology Research Laboratory at RPCI, and leads a number of research programs focused on thoracic cancers.

**Dr. Santosh Patnaik** is an Affiliate Member in the Department of Thoracic Surgery at the Roswell Park Cancer Institute in Buffalo, New York, USA. He received his medical school degree from the All India Institute of Medical Sciences in New Delhi, and his doctoral degree from the Albert Einstein College of Medicine in Bronx, New York, USA. His research centers around the biology and applications of microRNAs.
